# *Echinococcus multilocularis* in North America: the great unknown[Fn FN1]

**DOI:** 10.1051/parasite/2014069

**Published:** 2014-12-23

**Authors:** Alessandro Massolo, Stefano Liccioli, Christine Budke, Claudia Klein

**Affiliations:** 1 Department of Ecosystem and Public Health, Faculty of Veterinary Medicine, University of Calgary Calgary Alberta Canada; 2 Department of Biological Sciences, Faculty of Sciences, University of Calgary Calgary Alberta Canada; 3 Department of Veterinary Integrative Biosciences, College of Veterinary Medicine & Biomedical Sciences, Texas A&M University College Station Texas USA; 4 Department of Veterinary Clinical and Diagnostic Sciences, Faculty of Veterinary Medicine, University of Calgary Calgary Alberta Canada

**Keywords:** *Echinococcus multilocularis*, North America, Alveolar Echinococcosis, Domestic hosts, Wild hosts

## Abstract

Over the last decade, studies have begun to shed light on the distribution and genetic characterization of *Echinococcus multilocularis*, the causative agent of alveolar echinococcosis (AE), in North America. Recent findings indicate that the parasite is likely expanding its range in the central region of the United States and Canada and that invasions of European strains might have occurred. In our review, we present the available data on *E. multilocularis* infections in wild and domestic animals and humans in North America and emphasize the lack of knowledge on the distribution of the parasite in wild and domestic hosts. Furthermore, we stress the need to better understand the complexity of host communities and their roles in shaping the transmission and distribution of the parasite. We hypothesize that a lack of knowledge about AE by North American physicians might result in the misdiagnosis of cases and an underestimation of disease incidence. The endemic presence of the parasite in urban areas and a recent human case in Alberta, Canada, suggest that the scientific community may need to reconsider the local public health risks, re-assess past cases that might have been overlooked and increase surveillance efforts to identify new cases of human AE.

## Abstract (fr)

Au cours des dernières décennies, un certain nombre d’études a commencé à apporter quelques éclaircissements sur la distribution et la caractérisation génétique d’*Echinococcus multilocularis*, l’agent causal de l’échinococcose alvéolaire (EA), en Amérique du Nord. D’après les résultats les plus récents, le parasite est en train d’étendre son territoire au sein des régions centrales des États-Unis et du Canada, et il semble que l’on assiste à une invasion par des souches européennes. Dans cette revue générale, nous présentons les données actuellement disponibles sur l’infection par *E. multilocularis* chez les hôtes sauvages et domestiques en Amérique du Nord, et soulignons notre manque de connaissance concernant la distribution du parasite entre ces hôtes sauvages et domestiques. De plus, nous insistons sur la nécessité de mieux comprendre la complexité des communautés d’hôtes et leur rôle respectif dans la transmission et la distribution du parasite. Nous faisons l’hypothèse que l’absence de connaissance de l’EA par les médecins nord-américains est à l’origine de diagnostics erronés et donc d’une sous-estimation de l’incidence de la maladie. La présence endémique du parasite dans les zones urbaines, et un cas humain récent dans l’Alberta, au Canada, suggèrent que la communauté scientifique aurait peut-être intérêt à reconsidérer les risques locaux pour la santé publique, à reprendre l’évaluation diagnostique de cas qui auraient pu être négligés par le passé, et à renforcer la surveillance pour identifier de nouveaux cas humains d’EA.

## Introduction

Alveolar Echinococcosis (AE), a zoonosis caused by the tapeworm *Echinococcus multilocularis*, is recognized as an important emerging parasitic disease in the northern hemisphere [21, 88], and recently ranked as the third most relevant food-borne parasitic zoonosis [23]. The disease is estimated to affect more than 18,000 people per year globally, with most cases located in Europe and Asia [84]. China is believed to have the largest number of AE cases, with one study estimating that Chinese cases comprise approximately 91% of the global burden [88]. A recent study on the genetic characterization of *E. multilocularis* mitochondrial DNA [62] identified three strains corresponding to the geographical regions of Asia, Europe, and North America [18]. In North America, two sub-strains have been reported (N1 in the tundra zone and N2 in the central region) [62], supporting previous reports that parasites from Alaska and the north-central states of Montana and North Dakota differed in the development of their larval stages [5, 72].

Locally acquired human AE in North America has always been considered very rare, most cases historically reported being due to extra-continental infections (i.e. patients with travel history outside North America), with the important exception of a historical hotspot of the Asian strain that was discovered in St. Lawrence Island, Alaska, in the 1940s [78, 92]. To date, only three human cases are believed to have been caused by the central region strain of *E. multilocularis*, the first one in 1928 (Manitoba, Canada; [38]), the second in 1977 (Minnesota, USA; [26]), and the most recent in 2013 (Alberta, Canada; personal communication, Kinga T. Kowalewska-Grochowska). Despite an increased awareness of echinococcosis and other neglected tropical diseases, it is still unknown if AE is truly a rare disease in North America or if cases are being misdiagnosed due to a lack of physician knowledge about the disease [9, 92].

This review focuses on the available data on the presence of *E. multilocularis* in wild and domestic hosts and humans in North America, with the specific objectives of (1) identifying knowledge gaps in the parasite’s spatial distribution in wild and domestic host species, (2) providing a summary of available data on human infections, (3) illustrating the genetic characterization of *E. multilocularis* in North America, and (4) discussing possible emerging public health risks.

## *Echinococcus multilocularis* in wild hosts

Since the 1950s, several studies have been carried out to establish the primary wildlife hosts of *E. multilocularis* in North America, focusing on the Alaskan peninsula, Minnesota, and North Dakota, USA (e.g. [52, 53, 58, 70]) as well as Alberta, Canada [35]. However, despite the relevance of the parasite worldwide, and the groundbreaking initial work conducted by Rausch and Leiby [24, 26, 42, 50–54, 67, 68, 70–73, 79], the distribution, ecology, and epidemiology of *E. multilocularis* in North America is still largely unknown. In North America, *E. multilocularis* has been reported in the northern tundra zone (NTZ) of Alaska and Canada, and in the north central region (NCR), including four Canadian Provinces (Alberta, Saskatchewan, Manitoba, and British Columbia) and 13 US states (North Dakota, South Dakota, Iowa, Minnesota, Montana, Wyoming, Nebraska, Illinois, Wisconsin, Indiana, Ohio, and Missouri, and more recently central and southwestern Michigan) [18, 40, 84, 85] (Table 1).

**Table 1.** *Echinococcus multilocularis* infections in wild hosts in North America. Strains and haplotype as described in Nakao et al. [62].

Reference	Location	Definitive host				Intermediate host		
		Species (prevalence; *n*)	Method	Strain	Haplotype	Species (prevalence; *n*)	Strain	Haplotype
Chalmers and Barrett 1974 [14]	Lethbridge, AB, Canada					*Peromyscus maniculatus* (n/a)		
Catalano et al. 2012 [13]	Calgary, AB, Canada	*Canis latrans* (20.5%; 83)	Intestine	n/a				
Liccioli et al. 2012, 2013 [55, 56]	Calgary, AB, Canada	*Canis latrans* (29.5%; 61)	Intestine					
		*Canis latrans* (21.4%; 385)	Feces			*Peromyscus maniculatus* (0.7%; 305)		
						*Microtus pennsylvanicus* (0.7%; 267)		
						*Mus musculus* (0%; 2)		
						*Myodes gapperi* (1.4%; 71)		
						*Sorex* sp. (0%; 296)		
						*Zapus princeps* (0%; 32)		
						*Spermophilus tridecemlineatus* (0%; 6)		
						*Tamias minimus* (0%; 4)		
						*Thomomys talpoides* (0%; 3)		
Holmes et al. 1971 [35]	Edmonton, AB, Canada					*Peromyscus maniculatus* (27.7%; 216)		
						*Myodes gapperi* (0%; 122)		
						*Microtus pennsylvanicus* (0%; 1)		
Catalano et al. 2012 [13]	Edmonton, AB, Canada	*Canis latrans* (62.5%; 8)	Intestine	n/a				
Gesy et al. 2014 [28]	Edmonton, AB, Canada	*Canis latrans* (n/a)	Intestine		E, F			
Gesy et al. 2013 [27]	Quesnel, BC, Canada	*Canis latrans* (37%; 27)	Intestine	E		*Peromyscus maniculatus* (0%; 72)		
		*Vulpes vulpes* (17%; 6)	Intestine	E		*Microtus pennsylvanicus* (0%; 59)	E	
Gesy et al. 2014 [28]	100 Mile House, BC, Canada					*Microtus pennsylvanicus* (0%; 59)		
						*Zapus hudsonius* (0%; 16)		
						*Sorex* spp. (0%; 7)		
Gesy et al. 2014 [28]	Quesnel, BC, Canada	*Canis latrans* (37%; 27)	Intestine		D, E, L, M			
		*Vulpes vulpes* (17%; 6)	Intestine					
Gesy et al. 2014 [28]	Sathu, NT, Canada	*Canis lupus* (8%; 73)	Intestine		A, G, H			
Gesy et al. 2014 [28]	SK, Canada	*Canis lupus* (24%; 17)	Intestine		A, I	*Peromyscus maniculatus* (5%; 783)		A, I, J, K
Gesy et al. 2014 [28]	Riding Mtn, MB, Canada	*Canis lupus* (67%; 3)	Intestine		A, E			
Gesy et al. 2014 [28]	Karrak Lake, NU, Canada	*Vulpes lagopus* (4.8%; 354)	Feces			*Myodes rutilus* (0%; 8)		
						*Lemmus trimucronatus* (0%; 37)		
						*Dicrostonyx groenlandicus* (0%; 72)		
Gesy et al. 2014 [28]	Bylot Island, NU, Canada	*Vulpes lagopus* (22%; 50)	Feces		A, E, N, O, P, Q			
Schurer et al. 2014 [80]	NT, Canada	*Canis lupus* (8.2%; 73)	Intestine	E				
Schurer et al. 2014 [80]	SK, Canada	*Canis lupus* (23.5%; 17)	Intestine	E				
Schurer et al. 2014 [80]	MB, Canada	*Canis lupus* (67%; 3)	Intestine	E				
Rausch et al. 1990 [70]	AK, USA	*Vulpes lagopus* (80%; 1579)	Intestine					
Kirk 2011 [44]	AK, USA	*Vulpes lagopus* (27%; 26)	Intestine	A2, A4				
Holt et al. 2005 [37]	Barrow, AK, USA					*Lemmus trimucronatus* (0.9%; 467)		
						*Dicrostonyx rubricatus* (0%; 17)		
Nakao et al. 2009 [62]	AK, USA					voles (*n/a*; *11*)	N1	
Nakao et al. 2009 [62]	Indiana, USA	*Vulpes vulpes* (n/a)	Intestine	N2				
Storandt et al. 2002 [85]	NE, USA	*Vulpes vulpes* (37.5%; 72)	Intestine					
		*Canis latrans* (0%; 31)	Intestine					
Storandt et al. 2002 [85]	KS, USA	*Vulpes vulpes* (0%; 22)	Intestine					
		*Canis latrans* (0%; 89)	Intestine					
Kritsky et al. 1977 [51]	WY, USA		Intestine			*Neotoma cinerea rupicola*		
Storandt et al. 2002 [85]	WY, USA	*Vulpes vulpes* (0%; 31)	Intestine					
Storandt and Kazacos 1993 [83]	IN, USA	*Vulpes vulpes* (22%; 71)	Intestine					
		*Canis latrans* (18.6%; 70)	Intestine					
Storandt and Kazacos 1993 [83]	IL, USA	*Canis latrans* (35%; 17)	Intestine					
Storandt and Kazacos 2012 [84]	MI, USA	*Vulpes vulpes* (4.1%; 97)	Intestine					
Ballard 1984 [2]	WI, USA	*Vulpes vulpes* (8.3%; 72)	Intestine					
		*Urocyon cinereoargenteus* (0%; 31)						
Carney and Leiby 1968 [12]	MN, USA	*Vulpes vulpes*	Intestine			*Peromyscus maniculatus*		
Leiby et al. 1970, 1972 [53, 54]	MN, USA	*Vulpes vulpes* (5%; 277)	Intestine			*Peromyscus maniculatus* (1.9%; 53)		
						*Microtus pennsylvanicus* (0%; 326)		
						*Mus musculus* (0%; 24)		
Ballard and Vande Vusse 1983 [3]	NE, IL, USA	*Vulpes vulpes* (NE: 27%, 36; IL: 10%, 40)	Intestine					
Leiby et al. 1970 [53]	SD, USA	*Vulpes vulpes* (0.4%; 222)	Intestine			*Peromyscus maniculatus* (1.3%; 234)		
		*Canis latrans* (0%; 29)	Intestine			*Microtus pennsylvanicus* (0%; 67)		
						*Mus musculus* (0%; 4)		
Schantz et al. 1995 [77]	SD, USA	*Vulpes vulpes* (0.45%; 222)	Intestine					
Hildreth et al. 2000 [34]	SD, USA	*Canis latrans* (44.4%; 9)	Intestine					
		*Vulpes vulpes* (74.5%; 137)	Intestine					
Leiby et al. 1970 [53]	ND, USA	*Vulpes vulpes* (13.9%; 830)	Intestine			*Peromyscus maniculatus* (6%; 3335)		
		*Canis latrans* (6.3%; 111)	Intestine			*Microtus pennsylvanicus* (3.2%; 565)		
						*Mus musculus* (2.1%; 47)		
Rausch and Richards 1971 [72]	ND, USA	*Vulpes vulpes* (70%; 96)	Intestine			*Peromyscus maniculatus* (3%; 1080)		
Hildreth et al. 1991 [33]	ND, USA	*Vulpes vulpes* (90%; 45)	Intestine					
						*Microtus pennsylvanicus* (6%; 467)		
						*Sorex cinereus* (0%; 41)		
						*Blarina brevicauda* (0%; 70)		
						*Zapus hudsonius* (0%; 202)		
						*Reithrodontomys megalotis* (0%; 5)		
						*Mus musculus* (0%; 15)		
						*Myodes gapperi* (0%; 1)		
						*Citellus tridecemlineatus* (0%; 17)		
						*Citellus richardsoni* (0%; 1)		
Leiby et al. 1970 [53]	IA, USA	*Vulpes vulpes* (0.5%; 200)	Intestine			*Peromyscus maniculatus* (0.6%; 151)		
		*Canis latrans* (0%; 1)	Intestine			*Microtus pennsylvanicus* (5.5%; 36)		
						*Mus musculus* (0%; 4)		
Leiby et al. 1970 [53]	MT, USA	*Vulpes vulpes* (0%; 11)	Intestine			*Peromyscus maniculatus* (0.5%, 436)		
		*Canis latrans* (0%; 30)	Intestine			*Microtus pennsylvanicus* (0%; 39)		
						*Mus musculus* (0%; 12)		

The parasite is typically maintained in a sylvatic life cycle [22], with regional hosts determined by local predator-prey communities. In the NTZ, the parasite’s life cycle is sustained by the arctic fox (*Vulpes lagopus*) and its arvicoline rodent prey species such as the northern vole (*Microtus oeconomus*), the brown lemming (*Lemmus sibiricus*), and the northern red-backed vole (*Myodes rutilus*) [22]. Other carnivore species such as wolves (*Canis lupus*) may also harbor *E. multilocularis*, however, their role in parasite transmission has not been fully evaluated [80]. In the NCR, which is mostly characterized by prairie and boreal forests, the red fox (*Vulpes vulpes*) and the coyote (*Canis latrans*) are the primary definitive hosts, which prey on intermediate host species such as the deer mouse (*Peromyscus maniculatus*) and the meadow vole (*Microtus pennsylvanicus*) [35, 53]. The house mouse (*Mus musculus*) [51], the bushy tailed woodrat (*Neotoma cinerea*) [51, 53, 54], and the southern red-backed vole (*Myodes gapperi*) are other intermediate hosts of possible local importance [56].

Despite having a predominantly sylvatic life cycle, domestic animals can contribute to parasite transmission, resulting in the development of semi-synanthropic foci [22]. The most notable example of this, in North America, occurred in Native American communities on St. Lawrence Island, Alaska where domestic dogs were becoming infected by ingesting infected small mammals [77]. Another possible example is the case of two infected cats and an infected house mouse from a farm in North Dakota [54]. Although the authors speculated on the existence of a synanthropic cycle (cat – house mouse) of *E. multilocularis*, the alternative hypothesis that these latter cases were simply a spill-over from a sylvatic life cycle cannot be ruled out as no sampling from sylvatic hosts on the same farm was attempted, and the parasite was found in wild hosts no more than 13 km away from the farm. When a semi-synanthropic cycle is present, zoonotic transmission is probably more likely to occur given the close association of humans and domestic animals. Therefore, pet ownership can ultimately represent a risk factor for AE infection in certain circumstances [82]. Unfortunately, there has been a lack of consistency in the collection of data on *E. multilocularis* in North America. This lack of consistency is partially due to (1) variation in sampling designs, (2) the use of different diagnostic techniques with differing sensitivities and specificities, (3) spatial and temporal differences in data sources, and (4) regional differences in definitive and intermediate host species communities.

### Sampling design

Recent data from an urban area in North America indicate that the distribution of *E. multilocularis* is spatially and seasonally heterogeneous [57]. The frequent lack of an appropriate sampling design, which takes into account this spatio-temporal variability, makes longitudinal analyses unreliable. Moreover, while research conducted in Europe and Asia has indicated that interannual fluctuations of intermediate hosts are major drivers of *E. multilocularis* transmission [29, 30, 74], long-term studies in North America have mainly focused on density-dependent processes regulating rodent populations (e.g. [6, 47–49]). The available cross-sectional studies have estimated local prevalence in definitive and intermediate hosts (e.g. [13, 27, 35, 70]) in not well-defined host community systems. Therefore, trends in parasite transmission intensity and distribution over time are not well understood.

When studies are conducted over a short period of time (e.g. few months), it is not possible to take into account seasonal variations or the age structure of the host populations (e.g. [10]). Since it has been shown that significant variations in prevalence can be detected across different seasons (e.g. [10, 57]), surveys carried out over short periods of time and over small spatial scales cannot be compared to longer term studies carried out over large geographic areas. For example, *E. multilocularis* infection in intermediate hosts is believed to be highly clustered, resulting in prevalence as high as 10% in areas of only a few square meters, and less than 0.01% if calculated over larger areas in the same region [31]. For the above-mentioned reasons, it is necessary to consider different spatial and temporal scales to provide an adequate picture of the distribution of infections in a region, and be able to compare it with other regions or other time points. Finally, the origin of the samples can be a source of bias. For example, the use of hunted [36], trapped [85], or road-killed [13] animals may lead to prevalence estimates that are not representative of the overall population and will, therefore, not be comparable across studies.

### Diagnostic techniques

Even when the same study designs have been used (e.g., multiannual sampling of fox carcasses or feces), different diagnostic techniques with different sensitivities and specificities were often applied to detect *E. multilocularis* infections in definitive hosts. This is possibly related to the fact that studies reporting *E. multilocularis* prevalence are, in most cases, broad gastrointestinal parasite surveys (e.g. [36]) that use techniques that may not specifically target *E. multilocularis* infection. While there are methods available specifically for the detection of adult *Echinococcus* spp. [18], caution should still be used when comparing data obtained with different methodologies that have different sensitivity and different precision in counting worms per host.

The choice of diagnostic technique is important when examining definitive host feces [55]. Fecal examination and isolation of parasite eggs, for later molecular analysis, can be conducted using sugar flotation [28, 55] or centrifugation and sedimentation [57]. Although sugar flotation is commonly used for surveying gastrointestinal parasites in dogs and wild canids [75, 90], its sensitivity in detecting *E. multilocularis* in coyote feces was shown to be considerably lower than that reported for the ZnCl_2_ centrifugation and sedimentation technique (0.46 vs. 0.75) [57]. This latter protocol was specifically developed to detect *E. multilocularis* in fox feces [60], and is currently recommended for large-scale screening of canid hosts [17, 57].

Without data obtained with standardized techniques of known sensitivity, or without correcting for the differences in sensitivity, comparisons of prevalence between regions and across time intervals are likely to be misleading or uninformative. The technique adopted can also affect estimates of parasite intensity (sensu [11]). Diagnostic challenges may also occur when investigating prevalence in intermediate hosts. The investigation of macroscopic liver lesions in small mammals can result in an underestimation of prevalence [1], particularly when compared to histological and/or molecular tools which are better for the detection of early larval stage infections [10, 57].

### Temporal distribution of studies

The majority of the studies carried out in the NTZ are now relatively old [67, 68, 70], with research focusing on the epidemiology and risk factors associated with AE in highly endemic Native American communities in Alaska [77, 82, 93]. Although pioneer studies conducted in the NCR during the 1960s and 1970s [35, 36, 72, 76] were followed by some research effort in the 1990s and 2000s [34, 83, 85], large temporal gaps between studies make evaluation of trends very difficult, if not impossible. Moreover, the adoption of molecular diagnostic techniques in the 1990s [1, 20, 61] makes comparisons with older studies challenging [28, 57].

### Ecological communities

Differences in host communities and predator-prey relationships can affect *E. multilocularis* transmission. In the NTZ, the reliance of the arctic fox on arvicoline intermediate hosts as a food source [25] may be responsible for the high parasite prevalence observed in these definitive hosts [70], with surveys finding up to 100% of tested animals infected during a given season [24]. While coyotes and red foxes also rely on small mammals as a food source, their broader diet and more pronounced opportunism [16, 59] may partially explain why prevalence values tend to be lower in the central region, where these canid species act as the main definitive hosts. However, differences in temperature and humidity also likely affect parasite egg survival in the environment [91], and therefore, parasite transmission in the different regions [29]. Moreover, differences in host susceptibility to infection and the pathogenicity of different *E. multilocularis* strains [5] may also impact parasite transmission across eco-regions.

In summary, data available from North America are quite heterogeneous, making it difficult to identify trends in parasite distribution and abundance. While the hypothesis that the parasite’s range is expanding across North America fits with available information [39], further supportive data are required. Therefore, additional studies using comparable sampling designs and diagnostic techniques are needed.

## *Echinococcus multilocularis* in domestic hosts

### Dogs as definitive hosts

Both domestic dogs and cats can serve as definitive hosts for *E. multilocularis*, albeit cats seem to play an insignificant role in maintaining the life cycle [43]. However, unlike cats, dogs have also been reported to become infected with the larval stage of the parasite [41]. Limited information is available on the extent to which dogs act as definite hosts of *E. multilocularis* in North America since very few systematic studies have been carried out addressing this question. One notable exception is the north-western region of Alaska, in particular St. Lawrence Island, where *E. multilocularis* was found in the local dog population [22, 82, 93]. Here, on postmortem examination, the infection prevalence in dogs residing in specific Native American communities was up to 12% [77]. Consequently, owning a dog and living in close proximity to dogs was identified as a risk factor for human AE [82, 93].

To control AE on St. Lawrence Island, a control program focusing on the monthly administration of praziquantel to dogs was initiated. The prevalence of infection in intermediate hosts was used as a measure of success, with an 83% reduction in the prevalence in voles achieved over a 10-year period [69]. This decrease in intermediate host prevalence helped confirm the hypothesis that domestic dogs were contributing to the maintenance of the parasite life cycle. To the best of our knowledge, to date no other studies in North America on the prevalence in domestic dogs harboring adult *E. multilocularis* have been published, except for the ones from St. Lawrence Island, although the latter was a unique situation and not really comparable to the situation in the continent obtained within our laboratory. Based on the screening of 218 dog fecal samples from Calgary, Canada, using a modified centrifugation and sedimentation protocol followed by molecular confirmation [57], we estimated an *E. multilocularis* prevalence of approximately 0.46% (1/218). The positive dog was regularly walked in a known high endemic area in the city of Calgary (Alberta, Canada; [57]) and had a history of preying upon rodents (Massolo, Klein et al. unpublished data).

### Cats as definitive hosts

In North America, only a few studies have investigated the presence of *E. multilocularis* in cats. A study in Saskatchewan, Canada reported three positives out of 131 (2.29%) sampled free roaming cats [94]. The infected cats all had low intensity infections (30–50 adult worms). Around the same time, additional three cats were found to harbor adult *E. multilocularis* infections [54]. After finding an infected house mouse, in the same area, the existence of a possible domestic life cycle was hypothesized, but the alternative hypothesis that these domestic hosts were just spill-over cases was considered. However, although it is still controversial, it should be noted that cats are not optimal hosts for *E. multilocularis* and they are not believed to play a significant role in the parasite’s life cycle [43].

### Dogs as aberrant intermediate hosts

Rarely, dogs can develop AE with severe hepatic lesions. Recently, three dogs with AE were identified in Canada. One dog was identified in British Columbia [65], a second in Southern Ontario [64], and the third was found in Saskatoon (Audrey Tataryn, personal communication). The occurrence of these cases can either be an indicator of the spread of the disease or as a consequence of a heightened awareness of the disease following the first case report in 2012.

## Human infections with *Echinococcus multilocularis* in North America

While *E. multilocularis* is known to be endemic in northern regions of North America, there have been few human cases reported in the literature. The most widely documented cases, in this region, occurred on St. Lawrence Island, Alaska, USA starting in the 1940s [69]. In this endemic focus, working dogs were found to be eating infected small mammals and, subsequently, infecting humans. Concentrated control efforts aimed at dog deworming were able to eventually control this outbreak [93].

Besides the more than 70 cases reported on St. Lawrence Island, there have only been two locally acquired human AE cases from North America described in the literature. The first reported case was a 54-year-old fisherman from Winnipeg, Manitoba, Canada who presented with an abdominal mass in 1928 [38]. The patient was operated on in 1928, but succumbed to his disease in 1935. While this case was believed to be due to AE based on lesion morphology, no molecular confirmation was ever performed. The second reported case was a 56-year-old female from Minnesota, USA who presented for abdominal pain in 1977 [26]. In a 2008 publication, molecular evaluation of the cyst material obtained from the Minnesota case was shown to be almost identical to other isolates collected from intermediate hosts in South Dakota, USA [95]. A recent comparison of the mitochondrial gene cox1 sequence from an isolate from the Minnesota case with sequence data from adult worms collected from red fox in Indiana, USA [45] confirmed that the Minnesota case was infected with the North American N2 strain as described by Nakao and colleagues [62].

In May 2013, AE was reported in an immunosuppressed patient from Edmonton, Alberta, Canada, who had no history of travel (personal communication, Kinga T. Kowalewska-Grochowska). This case requires special consideration since immunosuppressed patients can be considered sentinels for locally emerging diseases. In a recent publication from France, a statistically significant increase in the number of immunosuppressed AE cases was described over the period 2002–2012 [15]. This report also indicated that the presence of immunosuppression may make AE cases more difficult to diagnose and treat.

**Figure 1.**
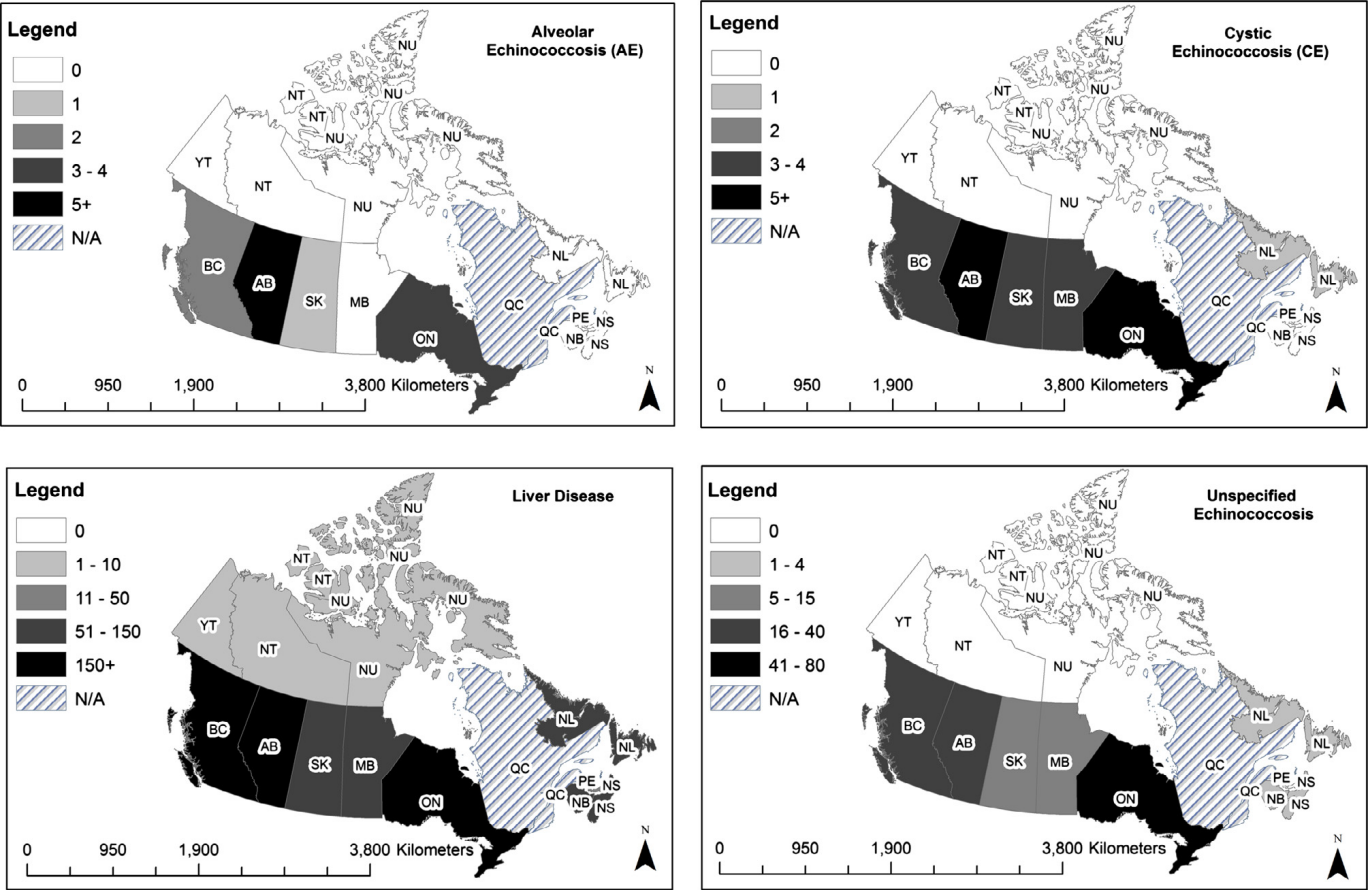
Distribution map by Province of Canadian hospital discharge data of Cystic Echinococcosis (CE), Alveolar Echinococcosis (AE), unspecified Echinococcosis and general liver diseases in Canada for the period 2001–2014. Data for Quebec were not available. Map by K. Berger, 2014.

A recent search of Canadian hospital discharge data, provided by the Canadian Institute for Health Information (CIHI) for the years 2001–2014, identified 242 hospital discharges with International Classification of Diseases-version 9 (ICD-9) codes for cystic echinococcosis (*n* = 39), alveolar echinococcosis (*n* = 12), or unspecified echinococcosis (*n* = 191) (Table 2, Fig. 1). These data indicate that Canadian healthcare providers have been treating cases of echinococcosis for many years. However, from this type of data, it is not possible to differentiate cases that were acquired locally from those that were acquired outside of North America. Large population centers, with considerable immigrant populations from known endemic regions, are likely to be areas with the highest numbers of identified cases. Considering the high level of immigration in North America, most of these cases are likely to have been acquired on other continents, and not represent autochthonous infections (e.g. [66]). This is not uncommon for countries characterized by high immigration rates. For example, in a study conducted in Germany, only nine out of 65 cystic echinococcosis cases were of German origin [63]. In another German study, AE was diagnosed in a refugee from an endemic area (e.g. [86]).

**Table 2.** Canadian hospital discharge data on Cystic Echinococcosis (CE), Alveolar Echinococcosis (AE), unspecified Echinococcosis and unspecified liver diseases for the period 2001–2014 by year and province. Data from Quebec were not available.

Year	CE	AE	Unspecified Echinococcosis	Unspecified Liver disease
2001–2002	1	0	4	64
2002–2003	0	1	11	199
2003–2004	3	0	10	150
2004–2005	7	0	7	164
2005–2006	3	4	16	152
2006–2007	4	3	19	179
2007–2008	3	0	18	181
2008–2009	1	0	18	143
2009–2010	3	2	10	150
2010–2011	7	1	24	136
2011–2012	3	0	21	153
2012–2013	2	1	17	160
2013–2014	2	0	16	118
*Canadian Province				
AB	11	5	37	163
BC	3	2	40	568
MB	4	0	14	138
NB	0	0	1	103
NL	1	0	3	58
NS	0	0	4	119
NT	0	0	0	5
NU	0	0	0	1
ON	17	4	79	644
PE	0	0	0	37
SK	3	1	13	105
YT	0	0	0	8
Total	39	12	191	1949

* AB-Alberta, BC-British Columbia, MB-Manitoba, NB-New Brunswick, NL-Newfoundland and Labrador, NS-Nova Scotia, NT-Northwest Territories, NU-Nunavut, ON-Ontario, PE-Prince Edward Island, SK-Saskatchewan, YT-Yukon. Data not available for Quebec.

Based on ICD-9 data alone, it is not possible to assess how many individuals the 242 hospital discharges actually represent, or to confirm the provided diagnosis. In addition, the infecting species was recorded for only a small proportion of the hospital discharges. That said, for the last decade, there has been an average of 19 discharges per year at hospitals located in all Canadian provinces except for Quebec, with no obvious upward or downward trend in case numbers. When the number of hospital discharges coded for unspecified liver disease was evaluated, for the same time period, there also did not appear to be an upward or downward trend in these case numbers.

A review of the available human data from North America indicates that there continues to be little known about the impact of human AE on this region. A lack of official reporting mechanisms means that, if cases are occurring, they are not being centrally recorded. In addition, it is possible that physicians in Canada and the USA may be misdiagnosing cases due to lack of training about the disease and its presentation. Therefore, a retrospective study evaluating banked liver tissue samples available from health service agencies (e.g., the Alberta Provincial Laboratory for Public Health in Alberta, Canada) would be helpful in assessing if cases of hepatic AE are being misdiagnosed [81]. Establishment of multidisciplinary teams composed of public health personnel, physicians, veterinarians, wildlife biologists, laboratory personnel, molecular biologists, epidemiologists, and ecologists is needed to develop a current picture of *E. multilocularis* transmission and the human health impact of AE on North America.

## Distribution of strains: the effect of globalization?

Information on the distribution of different *E. multilocularis* genotypes is needed to assess possible introductions that might have occurred in the last decades [18], as well as to evaluate the public health risks associated with different genotypes. Since the 1990s, isolates obtained from definitive and intermediate hosts originating from distinct geographical regions have been analyzed with respect to genetic differences. Analyzing a 471 base pair region of NADH dehydrogenase 1, Bowles and McManus [7] identified two genotypes (M1 and M2). M1 was identified in specimens from China, Alaska, and central North America, while M2 was found in a single European isolate. Based on polymorphism of microsatellites in the U1 snRNA gene of 41 isolates, Bretagne and co-workers described three distinct profiles of which profile A was found in all isolates from Europe, profile B was found in isolates from Alaska and Japan, and profile C was associated with isolates from Alaska and Montana [8]. Haag and co-workers analyzed the variance of two coding and two non-coding regions of the *E. multilocularis* genome [32] in 33 isolates and found minor differences in the intron of a homeobox-containing gene. Genotype A was found worldwide, whereas genotype B was restricted to St. Lawrence Island, Alaska.

The tandem repeat multilocus microsatellite EmsB exhibits a high genetic polymorphism, resulting in this genetic marker having a higher discriminatory power compared to others. Twenty-nine distinct genotypes have been identified [4, 46]. Nakao et al. described the division of 76 isolates into three distinct branches based on their geographical origin (Europe, Asia, or North America). Within the European, Asian, and North American clusters, 5, 10, and 2 haplotypes were identified, respectively [62]. To our knowledge, no isolates from autochthonous North American AE cases have been genotyped.

In 2009, a new haplotype was discovered which has similarities to previously described European haplotypes [27]. This new haplotype was found in foxes and coyotes from rural sites in three different Canadian Provinces (British Columbia, Alberta, and Saskatchewan). Thus far, this new haplotype has not been found in intermediate hosts. More recently, Massolo, Klein, and colleagues (unpublished data) found that this newly identified European strain was circulating in both urban (Calgary and Edmonton, Alberta, Canada) and rural environments. This poses interesting questions on the spread of this European strain into an area of known endemicity for the N2 strain and the possible public health risks.

The presence of this European strain in western Canada might be explained by two hypotheses. The first hypothesis is that the appearance of this new strain occurred as a consequence of introducing foxes from Europe for commercial use [18, 19]. The second hypothesis is that the parasite was introduced with dogs imported from Europe. Canada does not require treatment of dogs with praziquantel before entering the country. A recent risk assessment on importation of dogs infected with *E. multilocularis* into the United Kingdom pointed out that without the required treatment of dogs with praziquantel, it would be impossible to prevent the introduction of this parasite [87, 89]. The proposed hypotheses are not necessarily mutually exclusive, as they both could have occurred at different times and in different areas. At present, there is not enough information to support either hypothesis. However, the implementation of a praziquantel treatment policy for dogs being imported into North America would reduce the risk of introducing additional European strains.

## Conclusions and perspectives

Considering what is available in the literature on *E. multilocularis* ecology and epidemiology in North America, it is clear that there are substantial knowledge gaps. Available data are not spatially and temporally homogenous and differences in diagnostic methods and sampling design make comparisons difficult. Current hypotheses on parasite spread and the invasion of new strains are not yet sufficiently supported by data. In most cases, the presence of *E. multilocularis* in domestic animals is not monitored and its prevalence is not known even in settings where zoonotic transmission is more likely to occur (e.g., urban landscapes). The aggregation of thousands of pets in city parks and the presence of potentially infected coyotes, foxes, and small mammals should induce city managers and health authorities to assess the risk of transmission to dogs and humans. Despite their poor suitability as hosts for *E. multilocularis*, the role of cats as a potential route of infection to people should also be assessed. Reports of dogs acting as aberrant intermediate hosts have recently received increased attention in Canada, with these cases possibly representing a change in the relevance of *E. multilocularis* from an animal health standpoint.

The presence of a European strain of *E. multilocularis* in Canada, the discovery of a highly endemic area in Calgary, and the recent diagnosis of AE in an immunosuppressed patient in Edmonton suggest that we might expect to see an increase in the number of human cases in North America, similar to what has been seen in Central Europe [18]. Additional data are needed to help decide whether to develop new policies on wildlife management in urban settings and/or importation of dogs from areas where highly pathogenic strains of *E. multilocularis* are endemic. We feel that it is a priority to implement the following actions: (1) to assess the prevalence of *E. multilocularis* in wild and domestic hosts, in well-defined ecosystems, through long term studies with comparable sampling designs and diagnostic protocols, (2) to assess whether cases of human AE have been misdiagnosed through a retrospective analysis of biological samples available in the bio-banks of health service agencies, and finally (3) to develop a surveillance plan for human AE in North America. This would require the efforts of a multidisciplinary working group engaging parasitologists, ecologists, public health officers, pathologists, surgeons, veterinarians, epidemiologists, and others to increase preparedness and develop possible control and disease management strategies.

## References

[1] Al-Sabi MN, Jensen PM, Christensen MU, Kapel CM. 2013 Morphological and molecular analyses of larval taeniid species in small mammals from contrasting habitats in Denmark. Journal of Helminthology, ahead of print. 10.1017/S0022149X13000680 24160635

[2] Ballard NB. 1984 *Echinococcus multilocularis* in Wisconsin. Journal of Parasitology, 70, 844. 6512652

[3] Ballard NB, Vande Vusse FJ. 1983 *Echinococcus multilocularis* in Illinois and Nebraska. Journal of Parasitology, 69, 790–791. 6631648

[4] Bart JM, Knapp J, Gottstein B, El-Garch F, Giraudoux P, Glowatzki ML, Berthoud H, Maillard S, Piarroux R. 2006 EmsB, a tandem repeated multi-loci microsatellite, new tool to investigate the genetic diversity of *Echinococcus multilocularis*. Infection, Genetics and Evolution, 6, 390–400. 10.1016/j.meegid.2006.01.006 16504596

[5] Bartel MH, Seesee FM, Worley DE. 1992 Comparison of Montana and Alaska Isolates of *Echinococcus multilocularis* in Gerbils with observations on the cyst growth, hook characteristics, and host response. Journal of Parasitology, 78, 529–532. 1597801

[6] Boonstra R, Krebs CJ. 2012 Population dynamics of red-backed voles (*Myodes*) in North America. Oecologia, 168, 601–620. 21947547 10.1007/s00442-011-2120-z

[7] Bowles J, McManus DP. 1993 NADH dehydrogenase 1 gene sequences compared for species and strains of the genus *Echinococcus*. International Journal for Parasitology, 23, 969–972. 8106191 10.1016/0020-7519(93)90065-7

[8] Bretagne S, Assouline B, Vidaud D, Houin R, Vidaud M. 1996 *Echinococcus multilocularis*: microsatellite polymorphism in U1 snRNA genes. Experimental Parasitology, 82, 324–328. 8631384 10.1006/expr.1996.0040

[9] Budke CM, White ACJ, Garcia HH. 2009 Zoonotic larval cestode infections: neglected, neglected tropical diseases? PLoS Neglected Tropical Diseases, 3, e319. 19238190 10.1371/journal.pntd.0000319 PMC2638007

[10] Burlet P, Deplazes P, Hegglin D. 2011 Age, season and spatio-temporal factors affecting the prevalence of *Echinococcus multilocularis* and *Taenia taeniaeformis* in *Arvicola terrestris*. Parasites & Vectors, 4, 2–9. 21247427 10.1186/1756-3305-4-6 PMC3033848

[11] Bush AO, Lafferty KD, Lotz JM, Shostak AW. 1997 Parasitology meets ecology on its own terms: Margolis et al. revisited. Journal of Parasitology, 83, 575–583. 9267395

[12] Carney WP, Leiby PD. 1968 *Echinococcus multilocularis* in *Peromyscus maniculatus* and *Vulpes vulpes* from Minnesota. Journal of Parasitology, 54, 714. 5757663

[13] Catalano S, Lejeune M, Liccioli S, Verocai GG, Gesy KM, Jenkins EJ, Kutz SJ, Fuentealba C, Duignan PJ, Massolo A. 2012 *Echinococcus multilocularis* in urban coyotes (*Canis latrans*) in Alberta, Canada. Emerging Infectious Diseases, 18, 1625–1628. 23017505 10.3201/eid.1810.120119 PMC3471618

[14] Chalmers GA, Barrett MW. 1974 *Echinococcus multilocularis* Leuckart, 1863 in rodents in Southern Alberta. Canadian Journal of Zoology, 52, 1091. 4422509 10.1139/z74-145

[15] Chauchet A, Grenouillet F, Knapp J, Richou C, Delabrousse E, Dentan C, Millon L, Di Martino V, Contreras R, Deconinck E, Blagosklonov O, Vuitton DA, Bresson-Hadni S. 2014 Increased incidence and characteristics of Alveolar Echinococcosis in patients with immunosuppression-associated conditions. Clinical Infectious Diseases, 59, 1095–1104. 25034426 10.1093/cid/ciu520

[16] Cypher BL. 1993 Food item use by three sympatric canids in southern Illinois. Transactions of the Illinois State Academy of Science, 86, 139–144.

[17] Davidson RK, Oines O, Madslien K, Mathis A. 2009 *Echinococcus multilocularis*-adaptation of a worm egg isolation procedure coupled with a multiplex PCR assay to carry out large-scale screening of red foxes (*Vulpes vulpes*) in Norway. Parasitology Research, 104, 509–514. 18923842 10.1007/s00436-008-1222-y

[18] Davidson RK, Romig T, Jenkins E, Tryland M, Robertson LJ. 2012 The impact of globalisation on the distribution of *Echinococcus multilocularis*. Trends in Parasitology, 28, 239–247. 22542923 10.1016/j.pt.2012.03.004

[19] Davidson WR, Appel MJ, Doster GL, Baker OE, Brown JF. 1992 Diseases and parasites of red foxes, gray foxes, and coyotes from commercial sources selling to fox-chasing enclosures. Journal of Wildlife Diseases, 28, 581–589. 1474656 10.7589/0090-3558-28.4.581

[20] Dinkel A, Von Nikisch-Rosenegk M, Bilger B, Merli M, Lucius R, Romig T. 1998 Detection of *Echinococcus multilocularis* in the definitive host: coprodiagnosis by PCR as an alternative to necropsy. Journal of Clinical Microbiology, 36, 1871–1876. 9650927 10.1128/jcm.36.7.1871-1876.1998 PMC104943

[21] Eckert J, Deplazes P. 2004 Biological, epidemiological, and clinical aspects of echinococcosis, a zoonosis of increasing concern. Clinical Microbiology Reviews, 17, 107–135. 14726458 10.1128/CMR.17.1.107-135.2004 PMC321468

[22] Eckert J, Gemmell MA, Meslin F-X, Pawłowski ZS. 2001 WHO/OIE manual on echinococcosis in humans and animals: a public health problem of global concern. OIE/World Organisation for Animal Health: Paris, France 286 pp.

[23] FAO/WHO. 2014 Multicriteria-based ranking for risk management of food-borne parasites. Microbiological Risk Assessment Series. Food and Agriculture Organization of the United Nations/World Health Organization: Rome, Italy 302 pp.

[24] Fay FH, Rausch RL. 1964 The seasonal cycle of abundance of *Echinococcus multilocularis* in naturally infected arctic foxes, in First International Congress of Parasitology. Vol. 2, Pergamon Press: Oxford/New York p. 765–766.

[25] Fay FH, Stephenson RO. 1989 Annual, seasonal, and habitat-related variation in feeding habits of the arctic fox (*Alopex lagopus*) on St. Lawrence Island, Bering Sea. Canadian Journal of Zoology, 67, 1986–1994.

[26] Gamble WG, Segal M, Schantz PM, Rausch RL. 1979 Alveolar hydatid disease in Minnesota. First human case acquired in the contiguous United States. Journal of the American Medical Association, 241, 904–907. 762867 10.1001/jama.241.9.904

[27] Gesy K, Hill JE, Schwantje H, Liccioli S, Jenkins EJ. 2013 Establishment of a European-type strain of *Echinococcus multilocularis* in Canadian wildlife. Parasitology, 140, 1133–1137. 23714582 10.1017/S0031182013000607

[28] Gesy KM, Schurer JM, Massolo A, Liccioli S, Elkin BT, Alisauskas R, Jenkins EJ. 2014 Unexpected diversity of the cestode *Echinococcus multilocularis* in wildlife in Canada. International Journal for Parasitology: Parasites and Wildlife, 3, 81–87. 25161905 10.1016/j.ijppaw.2014.03.002 PMC4142260

[29] Giraudoux P, Craig PS, Delattre P, Bao G, Bartholomot B, Harraga S, Quere JP, Raoul F, Wang Y, Shi D, Vuitton DA. 2003 Interactions between landscape changes and host communities can regulate *Echinococcus multilocularis* transmission. Parasitology, 127, S121–S131. 15027609

[30] Giraudoux P, Pleydell D, Raoul F, Quere J-P, Wang Q, Yang Y, Vuitton DA, Qiu J, Yang W, Craig PS. 2006 Transmission ecology of *Echinococcus multilocularis*: What are the ranges of parasite stability among various host communities in China? Parasitology International, 55, S237–S246. 16361111 10.1016/j.parint.2005.11.036

[31] Giraudoux P, Raoul F, Afonso E, Ziadinov I, Yang Y, Li L, Li T, Quere JP, Feng X, Wang Q, Wen H, Ito A, Craig PS. 2013 Transmission ecosystems of *Echinococcus multilocularis* in China and Central Asia. Parasitology, 140, 1655–1666. 23734823 10.1017/S0031182013000644 PMC3806041

[32] Haag KL, Zaha A, Araujo AM, Gottstein B. 1997 Reduced genetic variability within coding and non-coding regions of the *Echinococcus multilocularis* genome. Parasitology, 115 (Pt 5), 521–529. 9368903 10.1017/s0031182097001649

[33] Hildreth MB, Johnson MD, Kazacos KR. 1991 *Echinococcus multilocularis*: a zoonosis of increasing concern in the United States. Compendium on Continuing Education for the Practicing Veterinarian, 13, 727–741.

[34] Hildreth MB, Sriram S, Gottstein B, Wilson M, Schantz PM. 2000 Failure to identify alveolar echinococcosis in trappers from South Dakota in spite of high prevalence of *Echinococcus multilocularis* in wild canids. Journal of Parasitology, 86, 75–77. 10701567 10.1645/0022-3395(2000)086[0075:FTIAEI]2.0.CO;2

[35] Holmes JC, Mahrt JL, Samuel WM. 1971 The occurrence of *Echinococcus multilocularis* Leuckart, 1863 in Alberta. Canadian Journal of Zoology, 49, 575–576. 5106581 10.1139/z71-090

[36] Holmes JC, Podesta R. 1968 The helminths of wolves and coyotes from the forested regions of Alberta. Canadian Journal of Zoology, 46, 1193–1204.

[37] Holt DW, Hanns C, O’Hara T, Burek K, Frantz R. 2005 New distribution records of *Echinococcus multilocularis* in the brown lemming from Barrow, Alaska, USA. Journal of Wildlife Diseases, 41, 257–259. 15827234 10.7589/0090-3558-41.1.257

[38] James E, Boyd W. 1937 *Echinococcus alveolaris* (with the report of a case). Canadian Medical Association Journal, 36, 354–356. 20320583 PMC1562016

[39] Jenkins DJ, Romig T, Thompson RCA. 2005 Emergence/re-emergence of *Echinococcus* spp. – a global update. International Journal for Parasitology, 35, 1205–1219. 16157340 10.1016/j.ijpara.2005.07.014

[40] Jenkins EJ, Peregrine AS, Hill JE, Somers C, Gesy K, Barnes B, Gottstein B, Polley L. 2012 Detection of European strain of *Echinococcus multilocularis* in North America. Emerging Infectious Diseases, 18, 1010–1012. 22608114 10.3201/eid1806.111420 PMC3358155

[41] Jones A, Pybus MJ. 2001 Taeniasis and Echinococcosis, in Parasitic Diseases of Wild Mammals (Second Edition), Samuel WM, Pybus MJ, Kocan AA, Editors. Iowa State University Press p. 150–192.

[42] Kagan IG, Norman L, Leiby PD. 1965 Biologic identification of Cestode *Echinococcus multilocularis* isolated from foxes in North Dakota. Journal of Parasitology, 51, 807–808. 5861363

[43] Kapel CMO, Torgerson PR, Thompson RCA, Deplazes P. 2006 Reproductive potential of *Echinococcus multilocularis* in experimentally infected foxes, dogs, raccoon dogs and cats. International Journal for Parasitology, 36, 79–86. 16199043 10.1016/j.ijpara.2005.08.012

[44] Kirk CM. 2011 Sentinels of Arctic ecosystem health: polar bear and arctic fox. PhD thesis, University of Alaska, Fairbanks.

[45] Klein C, Massolo A. 2014 The need of straintyping *Echinococcus multilocularis* in human cases of Alveolar Echinococcosis in North America – the N2 strain caused the case in Minnesota in 1977. American Journal of Tropical Medicine and Hygene, 17, 14–0484.

[46] Knapp J, Bart JM, Glowatzki ML, Ito A, Gerard S, Maillard S, Piarroux R, Gottstein B. 2007 Assessment of use of microsatellite polymorphism analysis for improving spatial distribution tracking of *Echinococcus multilocularis*. Journal of Clinical Microbiology, 45, 2943–2950. 17634311 10.1128/JCM.02107-06 PMC2045259

[47] Krebs CJ. 1996 Population cycles revisited. Journal of Mammalogy, 77, 8–24.

[48] Krebs CJ, Boonstra R, Boutin S, Sinclair AR, Smith JN, Gilbert BS, Martin K, O’Donoghue M, Turkington R. 2014 Trophic dynamics of the boreal forests of the Kluane Region. Arctic, 67 (Suppl. 1), 71–81.

[49] Krebs CJ, Reid D, Kenney AJ, Gilbert S. 2011 Fluctuations in lemming populations in north Yukon, Canada, 2007–2010. Canadian Journal of Zoology, 89, 297–306.

[50] Kritsky DC, Leiby PD. 1978 Studies on sylvatic echinococcosis. 5. Factors influencing prevalence of *Echinococcus multilocularis* Leuckart 1863, in red foxes from North Dakota, 1965–1972. Journal of Parasitology, 64, 625–634. 682065

[51] Kritsky DC, Leiby PD, Miller GE. 1977 The natural occurrence of *Echinococcus multilocularis* in the bushy-tailed woodrat, *Neotoma cinerea rupicola*, in Wyoming. American Journal of Tropical Medicine and Hygiene, 26, 1046–1047. 907044 10.4269/ajtmh.1977.26.1046

[52] Leiby P, Olsen O. 1964 The cestode *Echinococcus multilocularis* in foxes in North Dakota. Science, 145, 1066. 14172624 10.1126/science.145.3636.1066

[53] Leiby PD, Carney WP, Woods CE. 1970 Studies on sylvatic echinococcosis. III. Host occurrence and geographic distribution of *Echinococcus multilocularis* in the north central United States. Journal of Parasitology, 56, 1141–1150. 5534030

[54] Leiby PD, Kritsky DC. 1972 *Echinococcus multilocularis*: a possible domestic life cycle in central North America and its public health implications. Journal of Parasitology, 58, 1213–1215. 4641897

[55] Liccioli S, Catalano S, Kutz SJ, Lejeune M, Verocai GG, Duignan PJ, Fuentealba C, Hart M, Ruckstuhl KE, Massolo A. 2012 Gastrointestinal parasites of coyotes (*Canis latrans*) in the metropolitan area of Calgary, Alberta, Canada. Canadian Journal of Zoology, 90, 1023–1030.

[56] Liccioli S, Duignan PJ, Lejeune M, Deunk J, Majid S, Massolo A. 2013 A new intermediate host for *Echinococcus multilocularis*: the southern red-backed vole (*Myodes gapperi*) in urban landscape in Calgary, Canada. Parasitology International, 62, 355–357. 23608104 10.1016/j.parint.2013.03.007

[57] Liccioli S, Kutz SJ, Ruckstuhl KE, Massolo A. 2014 Spatial heterogeneity and temporal variations in *Echinococcus multilocularis* infections in wild hosts in a North American urban setting. International Journal for Parasitology, 44, 457–465. 24747533 10.1016/j.ijpara.2014.03.007

[58] Lubinsky G. 1957 List of helminths from Alberta Rodents. Canadian Journal of Zoology, 35, 623–627.

[59] MacCracken JG, Hansen RM. 1987 Coyote feeding strategies in southeastern Idaho: optimal foraging by an opportunistic predator? Journal of Wildlife Management, 51, 278–285.

[60] Mathis A, Deplazes P, Eckert J. 1996 An improved test system for PCR-based specific detection of *Echinococcus multilocularis* eggs. Journal of Helminthology, 70, 219–222. 8960218 10.1017/s0022149x00015443

[61] Monnier P, Cliquet F, Aubert M, Bretagne S. 1996 Improvement of a polymerase chain reaction assay for the detection of *Echinococcus multilocularis* DNA in faecal samples of foxes. Veterinary Parasitology, 67, 185–195. 9017867 10.1016/s0304-4017(96)01039-4

[62] Nakao M, Xiao N, Okamoto M, Yanagida T, Sako Y, Ito A. 2009 Geographic pattern of genetic variation in the fox tapeworm *Echinococcus multilocularis*. Parasitology International, 58, 384–389. 19651237 10.1016/j.parint.2009.07.010

[63] Orhun A, Müller-Stöver I, Holtfreter MC, Dedelen H, Häussinger D, Richter J. 2012 Epidemiologisch-klinische Charakteristika von Patienten mit zystischer Echinokokkose. Deutsche medizinische Wochenschrift, 137, 1039–1044. 22570097 10.1055/s-0032-1304951

[64] Peregrine A, Jenkins E, Gesy K, Kerr M, Scott S, Barnes B, Skelding A, Gottstein B. 2013 Emergence of *Echinococcus multilocularis* in dogs in Canada? in 24th International Conference of the World Association for the Advancement of Veterinary Parasitology, Perth, Australia.

[65] Peregrine AS, Jenkins EJ, Barnes B, Johnson S, Polley L, Barker IK, De Wolf B, Gottstein B. 2012 Alveolar hydatid disease (*Echinococcus multilocularis*) in the liver of a Canadian dog in British Columbia, a newly endemic region. Canadian Veterinary Journal, 53, 870–874. PMC3398525 23372195

[66] Pilsczek FH. 2011 Infectious diseases of Afghan immigrants in the United States: review of published reports. Journal of Ayub Medical College Abbottabad, 23, 159–162. 22830174

[67] Rausch R, Schiller EL. 1954 Studies on the helminth fauna of Alaska. XXIV. *Echinococcus sibiricensis* n. sp., from St. Lawrence Island. Journal of Parasitology, 40, 659–662. 13212541

[68] Rausch R, Schiller EL. 1956 Studies on the helminth fauna of Alaska. XXV. The ecology and public health significance of *Echinococcus sibiricensis* Rausch & Schiller, 1954, on St. Lawrence Island. Parasitology, 46, 395–419. 13378885 10.1017/s0031182000026561

[69] Rausch R, Wilson J, Schantz P. 1990 A programme to reduce the risk of infection by *Echinococcus multilocularis*: the use of praziquantel to control the cestode in a village in the hyperendemic region of Alaska. Annals of Tropical Medicine and Parasitology, 84, 239–250. 2222026 10.1080/00034983.1990.11812463

[70] Rausch RL, Fay FH, Williamson FSL. 1990 The ecology of *Echinococcus multilocularis* (Cestoda, Taeniidae) on St. Lawrence Island, Alaska, USA, II. Helminth populations in the definitive host. Annales de Parasitologie Humaine et Comparée, 65, 131–140. 2080830 10.1051/parasite/1990653131

[71] Rausch RL, Maser C, Hoberg EP. 1983 Gastrointestinal helminths of the cougar, *Felis concolor* L., in northeastern Oregon. Journal of Wildlife Diseases, 19, 14–19. 6682458 10.7589/0090-3558-19.1.14

[72] Rausch RL, Richards SH. 1971 Observations on parasite-host relationships of *Echinococcus multilocularis* Leuckart, 1863, in North Dakota. Canadian Journal of Zoology, 49, 1317–1330. 5119812 10.1139/z71-198

[73] Rinder H, Rausch RL, Takahashi K, Kopp H, Thomschke A, Loescher T. 1997 Limited range of genetic variation in *Echinococcus multilocularis*. Journal of Parasitology, 83, 1045–1050. 9406776

[74] Saitoh T, Takahashi K. 1998 The role of vole populations in prevalence of the parasite (*Echinococcus multilocularis*) in foxes. Researches on Population Ecology, 40, 97–105.

[75] Salb AL, Barkema HW, Elkin BT, Thompson RC, Whiteside DP, Black SR, Dubey JP, Kutz SJ. 2008 Dogs as sources and sentinels of parasites in humans and wildlife, northern Canada. Emerging Infectious Diseases, 14, 60–63. 18258078 10.3201/eid1401.071113 PMC2600154

[76] Samuel WM, Ramalingam S, Carbyn LN. 1978 Helminths in coyotes (*Canis latrans* Say), wolves (*Canis lupus* L.), and red foxes (*Vulpes vulpes* L.) of southwestern Manitoba. Canadian Journal of Zoology, 56, 2614–2617. 751709 10.1139/z78-351

[77] Schantz PM, Chai J, Craig PS, Eckert J, Jenkins DJ, MacPherson CNL, Thakur A. 1995 Epidemiology and control of hydatid disease, in *Echinococcus* and hydatid disease, Thompson RCA, Lymberg AJ, Editors. Wallingford Oxon OX10 8DE, CAB International: England, UK p. 233–331.

[78] Schantz PM, von Reyn CF, Welty T, Schultz MG. 1976 Echinococcosis in Arizona and New Mexico. Survey of hospital records, 1969–1974. American Journal of Tropical Medicine and Hygiene, 25, 312–317. 1259090 10.4269/ajtmh.1976.25.312

[79] Schiller EL. 1955 Studies on the Helminth Fauna of Alaska .26. Some observations on the cold-resistance of eggs of *Echinococcus sibiricensis* Rausch and Schiller, 1954. Journal of Parasitology, 41, 578–582. 13272110

[80] Schurer JM, Gesy KM, Elkin BT, Jenkins EJ. 2014 *Echinococcus multilocularis* and *Echinococcus canadensis* in wolves from western Canada. Parasitology, 141, 159–163. 24135428 10.1017/S0031182013001716

[81] Somily A, Robinson JL, Miedzinski LJ, Bhargava R, Marrie TJ. 2005 Echinococcal disease in Alberta, Canada: more than a calcified opacity. BMC Infectious Diseases, 5, 34. 15904502 10.1186/1471-2334-5-34 PMC1156894

[82] Stehr-Green JK, Stehr-Green PA, Schantz PM, Wilson JF, Lanier A. 1988 Risk factors for infection with *Echinococcus multilocularis* in Alaska. American Journal of Tropical Medicine and Hygiene, 38, 380–385. 3354771 10.4269/ajtmh.1988.38.380

[83] Storandt ST, Kazacos KR. 1993 *Echinococcus multilocularis* identified in Indiana, Ohio, and east-central Illinois. Journal of Parasitology, 79, 301–305. 8459347

[84] Storandt ST, Kazacos KR. 2012 *Echinococcus multilocularis* identified in Michigan with additional records from Ohio. Journal of Parasitology, 98, 891–893. 22339082 10.1645/GE-3057.1

[85] Storandt ST, Virchow DR, Dryden MW, Hygnstrom SE, Kazacos KR. 2002 Distribution and prevalence of *Echinococcus multilocularis* in wild predators in Nebraska, Kansas, and Wyoming. Journal of Parasitology, 88, 420–422. 12054030 10.1645/0022-3395(2002)088[0420:DAPOEM]2.0.CO;2

[86] Tappe D, Weise D, Ziegler U, Muller A, Mullges W, Stich A. 2008 Brain and lung metastasis of alveolar echinococcosis in a refugee from a hyperendemic area. Journal of Medical Microbiology, 57, 1420–1423. 18927422 10.1099/jmm.0.2008/002816-0

[87] Torgerson PR, Craig PS. 2009 Risk assessment of importation of dogs infected with *Echinococcus multilocularis* into the UK. Veterinary Record, 165, 366–368. 19783849 10.1136/vr.165.13.366

[88] Torgerson PR, Keller K, Magnotta M, Ragland N. 2010 The global burden of Alveolar Echinococcosis. PLoS Neglected Tropical Diseases, 4, e722. 20582310 10.1371/journal.pntd.0000722 PMC2889826

[89] Toth B, Frost A, Roberts H. 2010 The change in likelihood of *Echinococcus multilocularis* (Alveolar Echinococcosis) introduction into the United Kingdom as a consequence of adopting the existing harmonised Community rules for the non-commercial movements of pet animals. Version 2.0 ed. Defra: London 29 pp.

[90] Unruh D, King J, Allen J, Eaton R. 1973 Parasites of dogs from Indian settlements in northwestern Canada: a survey with public health implications. Canadian Journal of Comparative Medicine, 37, 25. 4265550 PMC1319720

[91] Veit P, Bilger B, Schad V, Schaefer J, Frank W, Lucius R. 1995 Influence of environmental factors on the infectivity of *Echinococcus multilocularis* eggs. Parasitology, 110, 79–86. 7845716 10.1017/s0031182000081075

[92] WHO Informal Working Group on Cystic and Alveolar Echinococcosis Surveillance PaC, Editor. 2011 Report of the WHO Informal Working Group on Cystic and Alveolar Echinococcosis Surveillance, Prevention and Control, with the Participation of the Food and Agriculture Organization of the United Nations and the World Organisation for Animal Health. Department of Control of Neglected Tropical Diseases, World Health Organization: Geneva, Switzerland 21 pp.

[93] Wilson JF, Rausch RL. 1980 Alveolar hydatid-disease – a review of clinical-features of 33 indigenous cases of *Echinococcus multilocularis* infection in Alaskan Eskimos. American Journal of Tropical Medicine and Hygiene, 29, 1340–1355. 7446824

[94] Wobeser GA. 1971 The occurrence of *Echinococcus multilocularis* (Leukart, 1863) in cats near Saskatoon, Saskatchewan. Canadian Veterinary Journal, 12, 65–68. PMC1695256 5104959

[95] Yamasaki H, Nakao M, Nakaya K, Schantz PM, Ito A. 2008 Genetic analysis of *Echinococcus multilocularis* originating from a patient with alveolar echinococcosis occurring in Minnesota in 1977. American Journal of Tropical Medicine and Hygiene, 79, 245–247. 18689631

